# Diagnostic accuracy of procalcitonin in critically ill immunocompromised patients

**DOI:** 10.1186/1471-2334-11-224

**Published:** 2011-08-24

**Authors:** Nicolas Bele, Michael Darmon, Isaline Coquet, Jean-Paul Feugeas, Stéphane Legriel, Nadir Adaoui, Benoît Schlemmer, Élie Azoulay

**Affiliations:** 1 AP-HP, Hôpital Saint-Louis, Medical ICU Department, 75010 Paris, France; University Paris-7 Paris-Diderot, UFR de Médecine, 75010 Paris, France; 2 Medical-Surgical Intensive Care Unit, Saint-Etienne university hospital, and Jean Monnet University, Avenue Albert Raymond, Saint-Etienne, 42270, France; 3 Thrombosis Research Group, EA 3065, Saint-Etienne University Hospital and Saint-Etienne Medical School, Avenue Albert Raymond, Saint-Etienne, 42270, France; 4 AP-HP, Hôpital Saint-Louis, Biochemistry Department, 75010 Paris, France; University Paris-7 Paris-Diderot, UFR de Médecine, 75010 Paris, France

**Keywords:** bacterial infection, neutropenia, HIV infection, immune deficiency, bone marrow transplantation, Sensitivity and Specificity.

## Abstract

**Background:**

Recognizing infection is crucial in immunocompromised patients with organ dysfunction. Our objective was to assess the diagnostic accuracy of procalcitonin (PCT) in critically ill immunocompromised patients.

**Methods:**

This prospective, observational study included patients with suspected sepsis. Patients were classified into one of three diagnostic groups: no infection, bacterial sepsis, and nonbacterial sepsis.

**Results:**

We included 119 patients with a median age of 54 years (interquartile range [IQR], 42-68 years). The general severity (SAPSII) and organ dysfunction (LOD) scores on day 1 were 45 (35-62.7) and 4 (2-6), respectively, and overall hospital mortality was 32.8%. Causes of immunodepression were hematological disorders (64 patients, 53.8%), HIV infection (31 patients, 26%), and solid cancers (26 patients, 21.8%). Bacterial sepsis was diagnosed in 58 patients and nonbacterial infections in nine patients (7.6%); 52 patients (43.7%) had no infection. PCT concentrations on the first ICU day were higher in the group with bacterial sepsis (4.42 [1.60-22.14] vs. 0.26 [0.09-1.26] ng/ml in patients without bacterial infection, *P *< 0.0001). PCT concentrations on day 1 that were > 0.5 ng/ml had 100% sensitivity but only 63% specificity for diagnosing bacterial sepsis. The area under the receiver operating characteristic (ROC) curve was 0.851 (0.78-0.92). In multivariate analyses, PCT concentrations > 0.5 ng/ml on day 1 independently predicted bacterial sepsis (odds ratio, 8.6; 95% confidence interval, 2.53-29.3; *P *= 0.0006). PCT concentrations were not significantly correlated with hospital mortality.

**Conclusion:**

Despite limited specificity in critically ill immunocompromised patients, PCT concentrations may help to rule out bacterial infection.

## Background

Procalcitonin (PCT), a peptide composed of 116 amino acids, is normally produced by the C cells in the thyroid gland. Because PCT is cleaved in the gland by a specific protease, circulating levels are very low (< 0.1 ng/ml) in healthy individuals. Serum PCT elevation in patients with bacterial sepsis was first reported in 1993 [1]. Over the last decade, PCT has gained ground as an early marker for bacterial sepsis in emergency departments and intensive care units (ICUs) [2-9]. The sensitivity and specificity of serum PCT for diagnosing bacterial sepsis was about 80% in most studies, and PCT performed better than CRP or other clinical or biological markers for sepsis [4,5,9]. Moreover, in unselected ICU patients, PCT predicted mortality [4,10-12]. More recent studies showed that PCT could be safely used to guide antibiotic use in lower respiratory tract infections or to shorten antibiotic treatment duration in immunocompetent patients with sepsis [13-16].

Immunocompromised patients can produce high serum PCT concentrations during bacterial sepsis [17-21]. However, few studies have evaluated the diagnostic performance of PCT in immunocompromised patients, and none have assessed the ability of PCT to diagnose bacterial sepsis in critically ill immunocompromised patients [22]. We conducted a prospective observational study to assess the accuracy of serum PCT levels for diagnosing bacterial sepsis in immunocompromised patients admitted to the ICU.

## Methods

The ethics committee of the French Society for Critical Care approved the study (SRLF-CE 07-188). This prospective observational study was conducted in the medical ICU of the Saint Louis teaching hospital in Paris (France) over a 6-mo period (February-July 2007). We included all immunocompromised patients defined as patients with any of the following: HIV infection (all stages), neutropenia (neutrophil count < 1 × 10^9^/L), exposure to glucocorticoids (> 0.5 mg/kg for > 30 d) and/or immunosuppressive or cytotoxic medications, solid organ transplantation, allogeneic or autologous stem cell transplantation, hematological malignancy, or solid tumor. Each of the included patients or next of kin received written information and give oral consent. Our IRB waived the need for written consent according to French Law.

Variables shown in Tables 1, table 2 and table 3 were recorded at baseline and on day 3. Hospital and 28-d survival was available for all patients. Severity of illness was measured using the Simplified Acute Physiology Score version II (SAPSII) [23] and the Logistic Organ Dysfunction score (LOD) [24].

**Table 1:** Patient characteristics at ICU admission

	Patients with Bacterial Infection n = 58	Patients without Bacterial Infection n = 61	Odds Ratio (95% confidence interval)	*P *value
Age (years)	62.4 (45.4-70.6)	46.3 (39.1-60.5)	1.03 (1.00-1.05)	0.153
Female gender - no. (%)	23 (39.7)	27 (44.3)	0.3 (0.39-1.72)	0.61
**Comorbidities**				
Liver disease, n (%)	2 (3.45)	4 (6.55)	1.96 (0.34-11.16)	0.44
Renal Dysfunction, n (%)	7 (12.07)	5 (8.19)	0.65 (0.19-2.18)	0.48
Diabetes mellitus, n (%)	6 (10.34)	1 (1.63)	0.14 (0.17-1.24)	0.07
COPD, n (%)	7 (12.07)	7 (11.47)	0.94 (0.31-2.88)	0.92
Heart disease, n (%)	7 (12.07)	7 (11.47)	0.94 (0.31-2.88)	0.92
**Reasons for ICU admission**				
Acute Respiratory Failure	29(50)	21(34.42)	0.52 (2.25-1.10)	0.08
Shock	25(43.1)	4(6.55)	10.79 (3.45-33.73)	**< 0.001**
Coma	1 (1.7)	15 (24.6)	0.54 (0.01-0.42)	**0.005**
Acute kidney injury	0 (0)	7 (11.47)	/	/
SAPSII score at admission	47.5 (38-66)	42 (30-54.5)	1.016/point (1.0-1.03)	**0.05**
LOD at Day 1	4 (3-6)	4 (1-6)	1.11 (0.32-1.01)	0.66
LOD at Day 3	3 (1-5)	2 (1-4)	1.1 (0.93-1.31)	0.28
**Invasive ventilation on Day 1**	20 (34.48)	27 (44.26)	0.66 (0.31-1.39)	0.27
**Vasoactive drugs on Day 1**	27 (46.55)	11 (18.03)	3.96 (1.72-9.10)	**0.002**
**Dialysis on Day 1**	7 (12.07)	6 (9.83)	1.26 (0.40-4.00)	0.69
Hospital mortality, n (%)	20 (34)	19 (31.15)	1.16 (0.54-2.50)	0.69

COPD, chronic obstructive pulmonary disease; LOD: Logistic Organ Dysfunction system;

SAPSII: Simplified Acute Physiology Score version II

**Table 2:** Clinical and laboratory characteristics on days 1 and 3

Patient Characteristics on days 1 and 3 Median (interquartile range)	Patients with Bacterial Infection n = 58	Patients without Bacterial Infection n = 61	Odds Ratio (95% confidence interval)	*P *value
**Clinical Characteristics**				
Body temperature, °C Day 1	38.5 (37.2-39.2)	37.5 (36.4-37.5)	1.37 (1.06-1.78)	**0.016**
Body temperature, °C Day 3	37.4 (37-38.2)	37.2 (36.6-37.6)	1.26 (0.84-1.91)	0.27
Leukocyte count, × 10^9^/L Day 1	4900 (1100-12800)	8200 (4500-13500)	1.0 (1.0-1.0)	0.13
Leukocyte count, × 10^9^/L Day 3	7900 (1900-15525)	7400 (3550-12942)	1.0 (1.0-1.0)	0.57
Platelet count, × 10^9^/L Day 1	92500 (21000-187000)	151000 (62000-277250)	1.0 (1.0-1.0)	**0.014**
Platelet count, × 10^9^/L Day 3	83000 (30000-217750)	115000 (53000-325000)	1.0 (1.0-1.0)	0.1
Neutrophil count, × 10^9^/L Day 1	3415 (300-8690)	4370 (2375-8320)	1.0 (0.99-1.0)	0.12
Fibrinogen (g/L) Day 1	5.06 (3.32-6.29)	3.90 (2.80-5.20)	1.19 (0.99-1.42)	0.052
Fibrinogen (g/L) Day 3	4.70 (3.82-7.35)	3.80 (3.02-5.60)	1.38 (1.0-1.0)	0.09
Lactate (mmol/L) Day 1	2.15 (1.3-4.5)	1.9 (1.37-2.83)	1.17 (0.99-1.38)	0.06
Lactate (mmol/L) Day 3	1.60 (1.23-2.25)	1.30 (0.97-1.78)	1.19 (0.69-2.05)	0.53
Procalcitonin (ng/ml) Day 1	4.42 (1.57-22.14)	0.26 (0.09-1.26)	1.05 (1.01-1.09)	**0.005**
Procalcitonin (ng/ml) Day 3	3.19 (1.17-16.13)	0.45 (0.10-1.69)	1.05 (1.01-1.09)	**0.03**

**Table 3:** Microbiogical finding in the 24 patients with microbiogically documented bacterial infection and the 9 patients with non-bacterial infection

	n = 34 (%)
**Bacterial infection**	**24 (70.6)**
*Positive blood culture *	20
Enterobacteria	13(38.2)
Pseudomonas Aeruginosa	5(14.7)
Stenotrophomonas maltophilia	1(2.9)
Listéria Monocytogenes	1(2.9)
Clostridium Spp	1(2.9)
Streptococcus	2(5.9)
Coagulase negative Staphylococcus	1(2.9)
**Non bacterial infection**	**9 (29.4%)**
***Fungal infection***	***5(14.7)***
Candida Albicans	1(2.9)
Aspergillus Fumigatus	1(2.9)
Pneumocystis Jirovecii	2(5.9)
Fusarium	1(2.9)
***Parasitic infection***	***3(8.8)***
Toxoplasmosis	3(8.8)
***Viral infection***	*1(2..9)*
Herpes simplex	1 (2.9)

Serum PCT was assayed on ICU days 1 and 3. Blood samples were centrifuged, decanted, aliquoted, and frozen at - 80°C. PCT was assayed using a time-resolved amplified cryptate emission (TRACE) technology assay (Kryptor PCT; BRAHMS, Hennigsdorf, Germany); the technicians who performed the TRACE assays were unaware of the results of the other tests. The assays were delayed and clinicians did not have access to the results during the study period.

The physicians in charge of the patients prescribed the microbiological tests and antimicrobial therapy according to usual practice in the ICU, without interference from the research team. The final diagnosis was established during a meeting of all of the ICU physicians after patient discharge. During this meeting, physicians were unaware of the PCT level. Patients were categorized as either having bacterial sepsis or not having a bacterial infection. For descriptive results, bacterial sepsis was classified as microbiologically documented when microorganisms were recovered from the infection site or blood, and as clinically documented when objective signs and symptoms of infection were found but cultures were negative. Similarly, patients without bacterial infections were categorized as having nonbacterial infections (fungal, parasitic, or viral) or a noninfectious condition.

### Statistical analysis

Results are reported as medians and quartiles (25th-75th percentile) or numbers and percentages. Patient characteristics were compared using the chi-square test or Fisher's exact test, as appropriate, for categorical variables, and the nonparametric Wilcoxon test or the Kruskal-Wallis test for continuous variables. A receiver-operating characteristic (ROC) curve was plotted for the ability of PCT levels to classify patients as having bacterial infection. A two-by-two table was established to determine the sensitivity and specificity for various PCT cutoff values.

To investigate associations between patient characteristics and diagnosis of bacterial sepsis, we first performed bivariate logistic regression analyses to look for a significant influence of each variable, as measured by the estimated odds ratio (OR) with a 95% confidence interval (95%CI). Variables yielding *P *values no greater than 0.20 in the bivariate analyses were entered into a multiple logistic regression model. We checked to ensure that omitting each of the selected variables induced no significant increase in likelihood.

All of the tests were two-sided, and *P *values smaller than 0.05 were considered statistically significant. Analyses were done using the SAS 9.1 software package (SAS Institute, Cary, NC, USA).

## Results

During the 6-mo study period, 320 patients were admitted to our ICU. One hundred and nineteen patients were included in this study; 98 (82.3%) of these were still in the ICU on day 3. Among the 21 other patients, 12 died and nine were discharged alive before day 3. Patient characteristics are reported in Tables 1 and 2. The reason for immunodepression was a hematological malignancy in 64 patients (53.8%), a solid tumor in 26 patients (21.8%), HIV infection in 31 patients (26%, including AIDS in 19 patients [16%]), and use of immunosuppressive agents in ten patients (8.4%). Of the included patients, 27 had neutropenia at ICU admission (22.7%) and eight were stem cell-transplant recipients (6.7%; allogeneic SCT in four patients).

**Figure 1:**
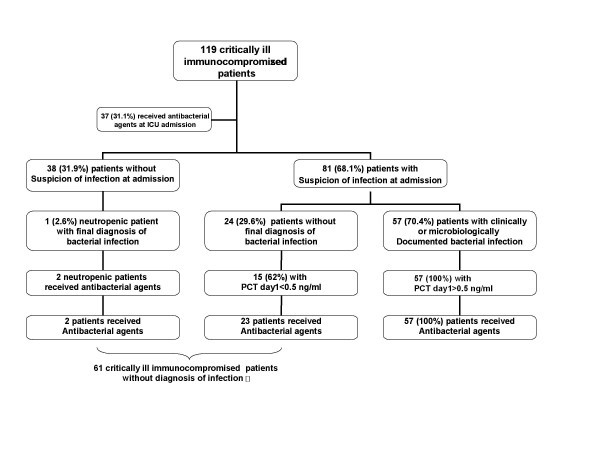
**Patient flow chart**.

The physicians suspected bacterial sepsis at ICU admission in 81 (68.1%) patients (Figure 1). Antimicrobials were started before ICU admission in 37 (31.1%) patients, and they were administered on the first ICU day to 82 (69%) patients.

The final diagnosis was clinically documented bacterial sepsis in 34 (28.6%) patients and microbiologically documented bacterial sepsis in 24 (20.2%) patients; thus, 58 (48.7%) patients had bacterial infections. Of the remaining patients, 61 patients had no bacterial infection, including nine patients with nonbacterial infections (7.6%) and noninfectious conditions in 52 (43.7%) patients. Of the patients with microbiologically documented bacterial infections, 20 (83.3%) had positive blood cultures (Table 3). Diagnoses in patients with noninfectious conditions were mainly malignant organ infiltration in 15 patients (28.9% of patients with noninfectious conditions), noninfectious neurological involvement in 11 patients (21.1% of patients with noninfectious conditions), cardiovascular events (including pulmonary embolism and cardiogenic pulmonary edema) in nine patients (17.3% of patients with noninfectious conditions), metabolic complications in nine patients (acute kidney injury or tumor lysis syndrome; 17.3% of patients with noninfectious conditions), and other noninfectious life threatening events in eight patients (15.4% of patients with noninfectious conditions).

**Figure 2:**
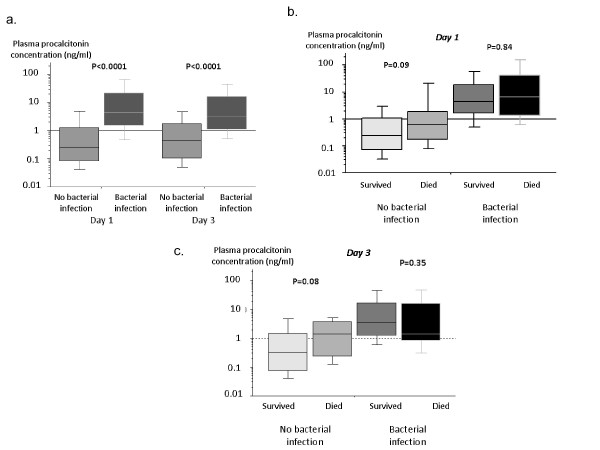
**Procalcitonin levels (ng/ml) in patients with bacterial infection and in the other patients on Day 1 and Day 3 (panel A) and in survivors and nonsurvivors (panel B)**.

Serum PCT concentrations on days 1 and 3 were significantly higher in patients with bacterial infections compared to all other patients (*P *< 0.0001; Figure 2, panel A). Patients with septic shock had higher serum PCT concentrations than patients who had severe sepsis without shock (15.18 ng/ml [4.17-48.81] vs 2.00 ng/ml [0.89-7.65]; *P *< 0.0001).

**Figure 3:**
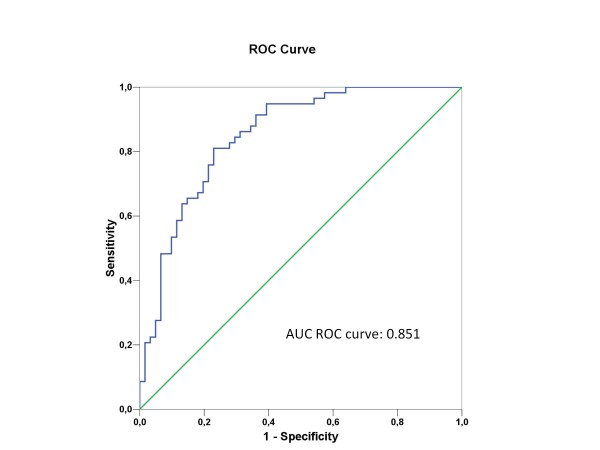
**ROC curve analysis of the performance of procalcitonin for diagnosing bacterial infection on the first day in the ICU**.

ROC curves are reported in Figure 3. The area under the curve (AUC) was 0.851 (95%CI 0.782-0.919). A cutoff value of 0.5 ng/ml was associated with 100% sensitivity but only 63% specificity. The performance of PCT at various cut-offs is reported in Table 4. In a multivariate analysis where bacterial infection was the outcome variable of interest, PCT concentrations of > 0.5 ng/ml independently predicted bacterial sepsis (Table 5).

**Table 4:** Performance of procalcitonin for detecting patients with bacterial infection at various cutoff values (± 95%CI)

PCT (ng/ml)	PCT > 0.5	PCT > 1.35	PCT > 2	PCT > 5
**Bacterial infection prevalence = 48.7%**				
Sensitivity	1.00 ± 0.00	0.81 ± 0.11	0.67 ± 0.12	0.50 ± 0.13
Specificity	0.63 ± 0.12	0.77 ± 0.11	0.82 ± 0.08	0.90 ± 0.08
				
Positive predictive value	0.72 ± 0.06	0.77 ± 0.10	0.78 ± 0.08	0.83 ± 0.15
Negative predictive value	1.00 ± 0.00	0.81 ± 0.11	0.72 ± 0.08	0.65 ± 0.08
				
Positive likelihood ratio	2.70	3.52	3.72	5.00
Negative likelihood ratio	0.00	0.25	0.40	0.56
				
Younden's index	0.63	0.58	0.49	0.40

**Table 5:** Multivariate analysis identifying independent predictors for bacterial infection

Variables	Odds ratio	95%CI	*P *Value
**Age, yr**	**1.05**	**1.01-1.09**	**0.006**
**Fever on D1**	**1.56**	**1.03-2.37**	**0.03**
**Shock on D1**	**8.37**	**2.06-34.1**	**0.003**
**Disseminated Intra-vascular Coagulation on D1**	**0.96**	**0.94-0.99**	**0.01**
**Procalcitonin serum level > 0.5 on D1 (ng/ml)**	**8.6**	**2.52-29.3**	**0.0006**
Fibrinogen serum level on D1 g/L	1.22	0.96-1.56	0.09

All of the 58 patients with clinically or microbiologically documented bacterial infections had PCT values above 0.5 ng/ml and all received antimicrobial agents. Among the 24 patients admitted with suspected bacterial infections but in whom bacterial infection was secondarily ruled out, 15 (62%) had PCT values < 0.5 ng/ml and all but one received antimicrobial agents. Including PCT in an algorithm for antimicrobial agent use with a sensitivity threshold of 0.5 ng/ml would have avoided antimicrobial therapy in 15 patients on day 1. Of the nine patients with nonbacterial infections, one patient had viral infection (PCT 4.3), five patients had fungal infections (median [IQR] PCT = 0.37, range 0.17-32.3) and three had parasitic infections (median PCT = 0.37, range 0.11-4.6). Multivariate analysis identifying independent predictors for bacterial infection are reported in table 5.

ICU and hospital mortality rates were 20.2% (24 deaths) and 32.8% (39 deaths), respectively. PCT concentrations on ICU days 1 and 3 were not significantly different in survivors and decedents (Figure 2, panel B). Independent predictors of hospital mortality (Table 6) were age (OR 1.03/y, 95%CI 1.001-1.06; *P *= 0.04) and invasive mechanical ventilation (OR 3.43, 95%CI 1.27-9.26; *P *= 0.01). Disseminated intravascular coagulation (DIC) at ICU admission was found to be protective against hospital mortality in the studied population (OR 0.96, 95%CI 0.94-0.99; *P *= 0.01).

**Table 6:** Multivariate logistic regression with hospital survival as the outcome variable of interest

Variables	Odds Ratio	95% CI	*P *Value
**Age, per year**	**1.03**	**1.001-1.061**	**0.04**
**Invasive mechanical ventilation**	**3.43**	**1.27-9.26**	**0.01**
			
Hemodialysis on D1	1.54	0.31-7.62	0.59
Lactate on D1, mmol/L	1.15	0.94-1.40	0.15
Procalcitonin on D1, ng/ml	1.0	0.98-1.01	0.83
Bacterial infection	0.52	0.17-1.6	0.25
**Disseminated intravascular coagulation on D1**	**0.97**	**0.94-0.99**	**0.02**

## Discussion

In this study, we evaluated the performance of PCT concentration as a marker for bacterial infection in immunocompromised patients admitted to the ICU. Higher PCT levels were not associated with hospital mortality. In addition, PCT concentrations were of limited value in diagnosing bacterial infection. Nevertheless, in our study population, PCT concentrations accurately ruled out a diagnosis of bacterial infection at a threshold of 0.5 ng/ml.

The absence of an association between PCT and mortality is in conflict with previously published studies in nonimmunocompromised patients, in which PCT levels at admission and daily thereafter correlated closely with hospital mortality [4,25,26]. This discrepancy can probably be ascribed to the impact of the underlying immunosuppression and organ dysfunctions on mortality. Regarding diagnostic performance, PCT levels on day 1 were significantly associated with bacterial infection. The area under the ROC curve was 0.851 (95% CI 0.782-0.919) and a cutoff of 0.5 ng/ml had 100% sensitivity. Similarly, in a study of immunocompromised patients with suspected pulmonary infection, serum PCT significantly predicted bacterial infection [21]. Moreover, the cutoff values found in our study are very close to those reported in nonimmunocompromised patients.

In nonimmunocompromised patients, serum PCT levels at admission and daily thereafter have been used to guide antibiotic prescriptions. PCT guidance substantially reduced antibiotic use in patients with lower respiratory tract infections and community-acquired pneumonia [13,14,26] and in patients admitted to the ICU with bacterial sepsis [16]. Our results suggest that PCT guidance might also be helpful in immunocompromised ICU patients.

Our study has several limitations. First, our population of immunocompromised patients was heterogeneous. While this diversity reflects everyday reality in ICUs, it may have led us to miss findings specific to particular subgroups. Moreover, 22% of patients had neutropenia upon ICU admission, a condition that requires special care when evaluating PCT. Second, the serum PCT level was measured on days 1 and 3. In most studies, PCT was measured at admission and a few hours later, allowing for a better assessment of the risk of bacterial infection. Our findings and earlier data support serial PCT measurement in patients who are not on antimicrobials. In addition, we included both microbiologically and clinically documented bacterial infection. Indeed, in the studied population, most of the included patients received antibiotics before ICU admission. This characteristic of the studied population may have limited the proportion of patients in whom microbiological documentation was possible. This choice may have induced a bias in evaluating the diagnostic performance of PCT. Similarly, the microbiological investigations were not standardized in the studied population, which also limits how our results can be interpreted. However, final classification of the patients was performed by physicians who were unaware of the PCT concentration. This may have limited the impact of the previously mentioned biases. Finally, bacterial infection is not the only factor that can lead to PCT elevation. Several solid malignancies, such as small-cell carcinoma of the lung and thyroid cancers, can spontaneously release PCT in the absence of bacterial infection. However, none of our patients had these types of tumors.

## Conclusion

PCT may help to rule out bacterial infection in immunocompromised patients admitted to the ICU. Although the performance of PCT concentrations as a diagnostic tool in this setting was limited, we believe that it should be investigated further in a larger study, and that an interventional study of antibiotic prescriptions guided by repeated PCT measurements in non-neutropenic immunocompromised patients is required.

## Competing interests statement

The authors declare that they have no competing interests.

## Authors' contributions

NB, MD and EA had full access to all of the data in the study and takes responsibility for the integrity of the data and the accuracy of the data analysis. ***Study concept and design: ***NB, EA, BS; ***Acquisition of data: ***NB, IC, JPF, SL, NA, EA ***Analysis and interpretation of data: ***NB, MD, EA; ***Drafting of the manuscript: ***NB, MD, EA; ***Critical revision of the manuscript for important intellectual content: ***NB, MD, IC, SL, NA, EA, JPF, EA, BS; ***Statistical analysis: ***MD, EA; The final version of the manuscript was approved by all of the authors.

## Pre-publication history

The pre-publication history for this paper can be accessed here:

http://www.biomedcentral.com/1471-2334/11/224/prepub
